# Relationship between Respiratory Morbidity and Environmental Exposure to Organochlorine Pesticides in Armenia

**DOI:** 10.5696/2156-9614-11.31.210904

**Published:** 2021-08-17

**Authors:** Natalya S. Tadevosyan, Gayane V. Kirakosyan, Susanna A. Muradyan, Susanna B. Poghosyan, Bavakan G. Khachatryan

**Affiliations:** Mkhitar Heratsi Yerevan State Medical University, Yerevan, Republic of Armenia

**Keywords:** organochlorine pesticides, pollution, environmental exposure, respiratory morbidity, endocrine disrupting chemicals

## Abstract

**Background.:**

Many studies have investigated the effects of organochlorine pesticides (OCPs) on adverse health outcomes. However, studies addressing the link between respiratory health and OCPs are limited. Organochlorine pesticides are stable compounds and belong to the class of endocrine disrupting chemicals that represent a threat to global health.

**Objectives.:**

The aim of the present study was to examine the association between respiratory morbidity and environmental exposure to OCPs in selected regions in Armenia.

**Methods.:**

The study was carried out in Lori and Gegharkunik provinces/marzes. The prevalence rate (per 100 000 population) and the average chronological indicators (ACh) for all respiratory diseases and asthma were calculated. Concentrations of OCPs (γ-hexachlorocyclohexane (γ-HCH), dichloro-diphenyl-trichloroethane [DDT], dichlorodiphenyldichloroethylene (DDE) and dichloro-diphenyl-dichloroethane (DDD)) were determined in soil and plant product samples and the average annual total concentration (AATC) of OCPs (γ-HCH + 4,4′-DDT + 4,4′-DDE+4,4′-DDD) was calculated.

**Results.:**

The ACI for all respiratory diseases showed a growth tendency in areas of Gegharkunik province ranging from 14.2 to 20.9% and an increase in asthma ranging from 9.4% to 174.6%. The highest levels of AATC of OCPs were found in soil sampled in Gegharkunik province: 9.48 ± 1.11 μg/kg and 8.10 ± 1.05 μg/kg and these levels differed significantly from those in Lori (p=0.01–0.0007). The AATC of OCPs in plant products from Gegharkunik was also statistically higher: 1.83±0.13 μg/kg, in comparison with that of Lori province 1.31±0.09 μg/kg (p = 0.001 – 0.0000).

**Conclusions.:**

The results indicate that the increased tendency of respiratory diseases and asthma could be related to OCP residues found in soil and plant products in Gegharkunik province. However, the role of OCPs should not be ignored. Further research is needed to study OCP contamination dynamics and clarify the role of OCPs in respiratory morbidity.

**Competing Interests.:**

The authors declare no competing financial interests.

## Introduction

Chronic respiratory diseases are a serious public health problem in all countries throughout the world. They account for four million deaths annually.^1^ An estimated 65 million people have moderate to severe chronic obstructive pulmonary disease (COPD), from which about 3 million die each year, making it the third leading cause of death worldwide and this number is growing.^2^ Chronic obstructive pulmonary disease accounts for 6% of all deaths worldwide each year.^2^ About 334 million people suffer from asthma, the most common chronic childhood disease, affecting 14% of children globally.^3^

Air pollution adversely affects the respiratory health of young populations due to the rapid lung development that occurs in this period.^4^–^6^ Environmental pollution results in the increase of chronic cough, rhinitis and infectious bronchitis.^7^,^8^ In children the most common chronic disease is asthma and this is significantly impacted by a number of environmental exposures.^9^ Research findings show that children living in rural areas are at a higher risk of airborne pesticide exposure and there are possible links between respiratory and allergic symptoms and this type of exposure.^9^

Pesticides have a unique place in the chemical pollution of the environment, as they are purposefully introduced into the environment and present a risk of negative impacts on both rural and general populations by increasing the prevalence of different diseases.^10^ Exposure to pesticides and different chemicals is an environmental determinant of lung cancer.^11^ Heavy metals and solvents along with pesticides have been associated with increasing rates of both asthma and allergies.^12^–^15^ Pesticides and, in particular, organochlorine pesticides (OCPs) are potentially hazardous to human health, due to their ability to have delayed effects at very low concentrations.

In recent decades, some OCPs have been classified as endocrine disrupting chemicals (EDCs).^16^–^18^ They have the ability to affect male and female reproductive function, endocrine function, the immune system, and increase susceptibility to various infections.^19^,^20^ In addition to pesticide exposure that might adversely affected lung function, many factors such as genetic, physiological status, as well as various environmental factors may also contribute to the development of respiratory symptoms, diseases, and deterioration of health.^21^

Persistent organic pollutants (POPs) are highly resistant to natural degradation processes. They are able to bioaccumulate and biomagnify, adversely affecting human health, and are a cause of immune system disorders, in particular increasing sensitivity to infections.^22^,^23^

Sunyer *et al.* (2010) reported that dichlorodiphenyldichloroethylene (DDE) and other organochlorines suppress immunity biomarkers in animals and humans.^23^ This immunologic suppression effect could explain the association observed between DDE and lower respiratory tract infection (LRTI).^23^ As LRTIs cause substantial morbidity in infancy, LRTI and wheeze are possible risk factors for subsequent childhood asthma.^24^,^25^ Disruption of the development of immune and respiratory systems by early-life exposure to POPs could result in reduced capacity to fight infections and increased risk of developing allergic manifestations later in life.^26^,^27^

**Abbreviations**
*AATC*: Average annual total concentration
*ACh*: Average chronological indicators
*EDC*: Endocrine disrupting chemicals
*USSR*: Union of Soviet Socialist Republics

Reduction of the risk of exposure to POPs is a very complicated task that requires common efforts from the worldwide community. This approach served as a basis for signing the “Convention on Persistent Organic Pollutants” (Stockholm Convention) to which the Republic of Armenia has been a party since 2003.^28^ Under the Convention provisions, the “National Implementation Plan for the Stockholm Convention on Persistent Organic Pollutants in the Republic of Armenia for 2016–2020” was developed and approved by the Governmental Decree of the Republic of Armenia. The National Implementation Plan includes a number of research studies which aim to monitor the levels of OCPs in the environment, their effects on humans, and aim to assess the risk to population health and the environment.^22^,^29^

Currently research aimed at the study of environmental health, pollution levels, especially by POPs/OCPs, and possible relationships between effects of various environmental factors and human health forms the scientific platform for primary prevention of environmental balance disturbances. In addition, it serves as a basis for the assessment of environmental risk factors for human health.^30^–^32^

Issues regarding the association between human health and environmental status are of high concern for Armenia as well. The Republic of Armenia is a mountainous country with limited resources of arable land. The country is located in the South Caucasus region and is bordered by Georgia to the north, Azerbaijan to the east, Iran to the south, and Turkey to the west and southwest. Armenia is characterized by a mountainous continental climate, which is very dry. The capital of Armenia is Yerevan. The permanent population was 2 959 700 in the beginning of 2020, 63.9% and 36.1% of which is urban and rural, respectively.^33^

Like all of the Republics of the former Union of Soviet Socialist Republics (USSR), OCPs were widely used in Armenia until 1986. The reason for such a large-scale application of OCPs was conditioned by the universal, multipurpose character of these compounds. Armenia has long had a well-developed agricultural economy. Until the 1980s, the republic practiced intense pesticide application. Total area load of pesticides averaged 9.0–35.5 kg per hectare. This value exceeded average levels of pesticide application in different regions of the former USSR.^34^ Agriculture remains one of the main sectors of the economy. Total agricultural lands are comprised by 2 044.2 thousand ha, of which 21.8% are mainly under grains and leguminous plants, potatoes and vegetables.^35^ In Armenia the load of OCPs per one hectare of arable lands was 8.4 kg and the load per capita was 1.72 kg. In the districts of the Ararat Valley, the load of OCPs per one hectare of arable lands was 21.1 kg and 5.3 kg, respectively. Studies conducted in different regions of the former USSR on OCPs levels in various environmental media served as the basis for banning the application of extremely hazardous OCPs in 1970 by the Order of Minister of Health of the former USSR.^22^

Statistical data show that non-communicable diseases, including chronic respiratory diseases such as COPD and bronchial asthma are the public health diseases of national importance in Armenia. In 2018, mortality rates from chronic diseases of the lower respiratory tract accounted for 2.38% of the 93% of total deaths reported to be caused by non-communicable diseases in Armenia.^36^ Stable negative dynamics of deaths from respiratory diseases were registered in 2013–2016 and, in particular, 1645 deaths were registered in 2013, of which 990 (60.2%) were deaths from chronic lower respiratory diseases. In 2016 the number of deaths was 2148, of which 1121 (52.2%) were attributed to chronic diseases of the lower respiratory tract, and in 2017 the number of deaths was 1644, of which 648 (39.4%) were attributed to chronic diseases of the lower respiratory tract.^36^

Mortality rates from respiratory diseases rank third in total mortality structure and has generally increased from year to year in proportion. Thus, in 2013 respiratory diseases accounted for 5.9% of mortality, 6.7% in 2014, 7.6% in 2016, and 6% in 2017. From 2013–2018 the incidence of COPD and bronchial asthma increased as well. In 2013, 20 530 cases were registered among the adult population, and in 2016 there were 23 570 cases, an increase of about 15%, while for the same period the number of sick persons diagnosed for the first time increased by about 32%.^36^

To our knowledge, the present study is one of the few to analyze the possible link between environmental exposure to OCPs and respiratory diseases and asthma, particularly in Armenia. The current study investigated levels of OCPs in soil and plant products in Lori and Gegharkunik marzes/provinces of the Republic of Armenia, which differ both economically and by population structure, and analyzed possible links with respiratory diseases and asthma through comparative analysis.

## Methods

**Figure 1:**
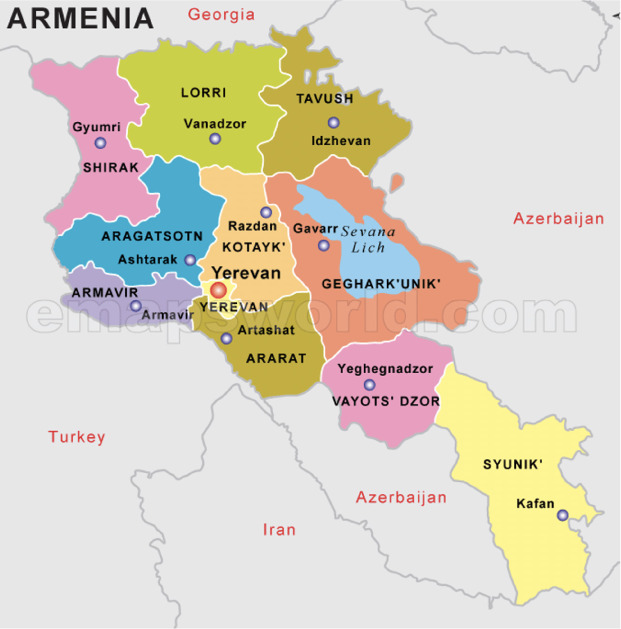
Map of the Republic of Armenia: provinces (marzes)

The study was implemented in three areas of Lori province: Spitak, Stepanavan, and Tashir and likewise in Gegharkunik province in Sevan, Gavar, Martuni, and Vardenis (*Figure 1*). The total population number did not differ significantly across provinces, with populations of 213 300 and 227 700 in Lori and Gegharkunik, respectively. These two provinces were selected due to their different economic development characteristics. Industry in Lori marz is primarily mining and manufacturing, making up 4.2% of the total Armenian economy. Likewise, in Lori marz, the agricultural sector comprises 8.2% of the total economy of Armenia. Lori marz is a more urbanized region with a population that is 59.1% urban (126 100 persons) and 40.9% rural (87 200 persons).^37^

Gegharkunik marz is a mainly agricultural region with a population that is 70.8% rural (161 100 persons) and 29.2% urban (66 600 persons). The local economy is primarily agricultural, particularly production of grain, potato, vegetable and animal husbandry products, representing 13.2% of the Armenian economy, while the industrial sector represents only 3.3% of the total economy.^37^

The provinces in the present study have different characteristics, both in terms of the structure of the economy and the proportion of rural population. Gegharkunik marz is characterized by a larger rural population (almost two-fold higher) and a high share of agriculture (1.6 times) in the overall economy than Lori marz.^37^

Morbidity data on respiratory disease and asthma from 2005–2015 in Lori and Gegharkunik were derived from reports of primary health care facilities submitted to the Ministry of Health of the Republic of Armenia. Prevalence rates (per 100 000 population) have been calculated according to the International Classification of Disease, 10^th^ Revision (ICD-10) for respiratory disease (ICD J00–J99) and asthma cases (ICD J45–J46) registered between 2005 and 2015.^38^ We used ICD 10 and not ICD 11 because ICD 10 is available in the Armenian language. Asthma prevalence rates were analyzed separately, because asthma is considered to be an environmental-related disease. The aforementioned rates served as additional criteria in the assessment of the effect of possible exposure to environmental contamination by agrochemicals on respiratory disease prevalence. To obtain a dynamic pattern of disease prevalence, the average chronological indicators (ACh) were calculated for total respiratory disease and asthma. Average chronological indicator is the average value calculated from time-varying values applied to analyze the dynamic series of prevalence rates of respiratory diseases over 10 years (2005–2015) in the following periods: 2005–2009 and 2010–2015. The calculation was done for each five-year period separately for each area studied.^39^

### Sampling sites

The study team identified five to six monitoring points in each province. Sampling the arable layer of cultivated areas was performed based on the agricultural work periods of spring (April–May), summer (June–July), and fall (September–October). Soil samples were collected from a depth of 0–25 cm by shovel which was washed by water after each sampling.

The envelope or cross-diagonal method was used for sampling in the present study. For each agricultural plot under study, at least one test site of at least 10 × 10 m was identified. Soil samples of 0.5 kg were taken from each corner and center (spot samples) of the test site (similar to an envelope). The spot samples were mixed to make a combined sample weighing at least 1.0 kg and placed in a plastic resealable bag numbered with the sampling point.^40^ In the study areas, five or six samples of soil were taken in each season. The number of samples varied depending on the sown areas under agricultural crops. Nine to 12 vegetable (potato, cabbage, carrot, beat, grain) and fruit (apple, pear, peach) samples were collected from the same cultivated field in each study area.

### Analysis

Concentrations of OCPs in soil and plant products sampled from 2013–2018 in the studied areas were analyzed by gas chromatography with electron-capture detection (Perkin-Elmer F-17, Great Britain) at the laboratory of Environmental Hygiene and Toxicology of Yerevan State Medical University. In Lori province, samples of soil and agricultural products were collected during the three agricultural seasons from 2013–2015 and in Gegharkunik sampling was done from 2016–2018. The following analytes were determined: γ-hexachlorocyclohexane (γ-HCH), dichloro-diphenyl-trichloroethane (4,4′-DDT), dichlorodiphenyldichloroethylene (4,4′-DDE) and dichloro-diphenyl-dichloroethane (4,4′-DDD) and the sensitivity of the method was 0.002–0.04 μg/kg.^41^ The average annual total concentration (AATC) of OCPs (γ-HCH + 4,4′-DDT + 4,4′-DDE + 4,4′-DDD) was calculated from the total sum of OCPs detected during the year across the three study seasons for soil and plant products, separately.

The results were expressed as mean ± SE. Data obtained on OCP values in the environmental media and plant production were processed and analyzed using standard software (Microsoft Excel). Comparisons between regions were performed using two-tailed Student's test for independent samples (t-test: two-sample assuming unequal variances). The level of statistical significance was estimated at p < 0.05.

## Results

**Figure 2:**
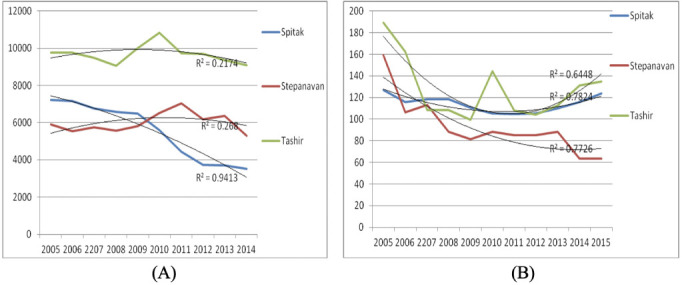
Prevalence of respiratory diseases (A) and asthma (B) cases in Lori province from 2005–2015, per 100,000 population

Data on the prevalence rates calculated for respiratory diseases (ICD J00–J99) and asthma (ICD J45–J46) registered in Lori marz from 2005 to 2015 are presented in Figure 2. A dynamic decrease in the respiratory disease was observed in Spitak area over the years (R^2^=0.94). In Stepanavan and Tashir, no significant changes in the rate of respiratory diseases were observed. A decrease in the rate of asthma was registered in Stepanavan (R^2^=0.77), from 159.3 in 2005 down to 63.7 in 2015. Meanwhile, no changes in asthma rates were observed in the Spitak (R^2^=0.78) and Tashir (R^2^=0.64) areas.

**Figure 3:**
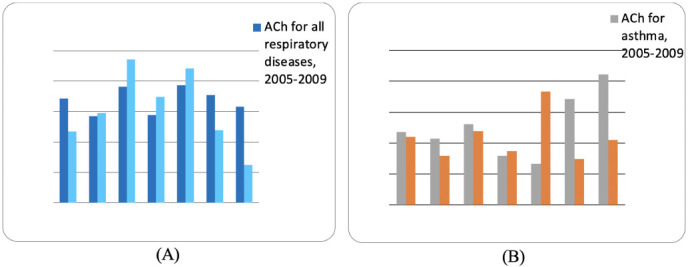
Average chronological (ACh) indicators for respiratory disease (A) and asthma (B) cases in Lori and Gegharkunik provinces from 2005–2015

The ACh for the respiratory diseases calculated for Spitak decreased by 31.6%, while in Stepanavan and Tashir, it increased by 3.95% and 23.5%, respectively. The ACh calculated for asthma cases in Spitak, Stepanavan and Tashir areas (Lori province) decreased by 6.7%, 25.6% and 8.95%, respectively *(Figure 3).*

**Figure 4:**
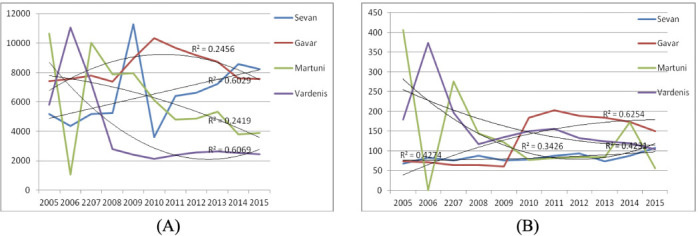
Prevalence of respiratory diseases (A) and asthma (B) cases in Gegharkunik Province from 2005–2015, per 100 000 population

The following pattern of prevalence rates was observed in Gegharkunik province *(Figure 4).* A tendency for dynamic increase in respiratory diseases was observed in the Sevan area over the study period: from 5166.4 in 2005 up to 8238.6 in 2015 (R^2^=0.60). In the Gavar area there was a small increasing tendency. A decrease in prevalence rates for respiratory diseases was recorded in Martuni (R^2^=0.24) and Vardenis (R^2^=0.61) over the study period: from 10667.9 and 5795.3 in 2005 down to 3899.9 and 2450.8 in 2015, respectively. For asthma rates a dynamic growth trend was observed in the Gavar area: from 74.0 in 2005 up to 150.0 in 2015. In the Martuni and Vardenis areas, a downward trend was observed: decreasing from 406.3 and 178.8 in 2005 down to 56.2 and 104.0 in 2015, respectively.

The ACh for respiratory diseases registered in Gegharkunik province increased in Sevan and Gavar areas by 20.9% and 14.2%, respectively, while in Martuni and Vardenis areas these indices decreased by 32.7% and 60.6%, respectively. The data obtained for asthma cases once again showed an increasing tendency recorded in Sevan and Gavar by 9.4% and 174.6% and a downward trend in Martuni and Vardenis areas of 56.5% and 50.0%, respectively (*Figure 2*).

The residues of certain OCPs were measured in soil and plant products sampled in the study areas and the average AATC of OCPs (HCH + DDT + DDE + DDD) was calculated (*Table 1*). The results of the analysis of environmental status in Gegharkunik province found that the AATC of OCPs in the soil sampled in the Martuni area was 4.99 ± 0.47 μg/kg, while in the Vardenis and Sevan/Gavar areas these levels were significantly higher: 9.48 ± 1.11 μg/kg and 8.10 ± 1.05 μg/kg, p = 0.001 and p = 0.02, respectively. The AATC of OCPs in plant products of Sevan/Gavar: 2.23 ± 0.11 μg/kg was also relatively higher in comparison with the Vardenis and Martuni areas: 1.70 ± 0.25 μg/kg and 1.78 ± 0.15 μg/kg, respectively, with statistical significance (p = 0.06 and p = 0.02, respectively).

**Table 1:** Organochlorine Pesticides in Soil and Plant Products of Lori and Gegharkunik

Province/area		OCPs in soil, μg/kg	OCPs in plant products, μg/kg
Lori	Spitak	7.3	0.82
	Stepanavan	4.63	1.75*
	Tashir	6.28	1.3*
Gegharkunik	Sevan-Gavar	8.1**	2.23**
	Martuni	4.99	1.78
	Vardenis	9.48**	1.7

Abbreviation: OCPs- Organochlorine pesticides

Note:

^*^differences statistically significant for Spitak, p = 0.0002–0.0003;

^**^differences statistically significant for Martuni, p = 0.001-0.02.

For Lori province, a relatively high AATC for OCPs was found in soil samples from the Spitak area: 7.30 ± 1.20 μg/kg compared to Tashir: 6.28 ± 0.38 μg/kg (p=0.05) and Stepanavan areas 4.63 ± 0.48 μg/kg. The AATC of OCPs in plant products of the Spitak area was 0.82 ± 0.07 μg/kg. In Tashir and Stepanavan areas these levels were 1.30 ± 0.09 μg/kg and 1.75 ± 0.19 μg/kg, respectively, which was statistically different from Spitak: p = 0.0002 and p = 0.0003, respectively *(Table 1).*

Comparison of the AATC of OCPs in plant products in the studied provinces showed that the recorded values in Gegharkunik province (1.83±0.13 μg/kg) were statistically higher than in Lori (1.31±0.09 μg/kg, p = 0.001 – 0.0000). The actual levels of certain OCPs (HCH, DDT, DDE, and DDD) determined in the environmental matrices and plant products sampled in the study areas did not exceed the standards established for these objects by the decision of the Customs Union Commission at the Eurasian Economic Commission.^42^

## Discussion

A comprehensive analysis of the ACh for all respiratory diseases was carried out. The ACh dynamics, recorded from 2005 to 2015, showed growth in the following areas: Stepanavan (3.95%) and Tashir (23.5%), Sevan (20.9%) and Gavar (14.2%). In Spitak, Martuni, and Vardenis areas the ACI decreased by 31.6%, 32.7%, and 60.6%, respectively.

An analysis of asthma prevalence rates in studied areas found that the highest values of the ACh were recorded in Gegharkunik province. In the dynamics of the studied years (2005–2015), growth in the ACh of asthma cases, which is considered to be a disease associated with environmental quality, was recorded in Sevan and Gavar areas (Gegharkunik province), of 9.4% and 174.6%, respectively. In Spitak, Stepanavan, Tashir (Lori province), Martuni and Vardenis (Gegharkunik province), the ACh rates calculated for asthma decreased by 6.7%, 25.6%, 8.95% and 56.5%, 50.0%, respectively.

A comparative analysis of the AATC of OCPs in soil and plant agricultural products and the ACI calculated for the studied areas was applied to study the possible association between environmental contamination by OCPs and respiratory health. It was shown that the AATC of OCPs recorded in soil sampled in Sevan/Gavar and Vardenis (Gegharkunik) were relatively high, and statistically higher values in plant products were also recorded in Sevan/Gavar. These findings were accompanied with an increased prevalence of respiratory disease and asthma cases in Sevan/Gavar (Gegharkunik province). The AATC of OCPs determined in soil and samples of plant products were lower in Lori compared to Gegharkunik province and asthma prevalence rates were decreased for all studied areas in Lori province.

Exposure to pesticides may cause a wide range of acute or chronic (long-term) health effects. Prolonged exposures to low levels of pesticides, especially persistent ones, are considered a risk factor for developing asthma, exacerbating a previous asthmatic condition or even triggering asthma attacks by increasing bronchial hyper-responsiveness.^43^–^45^

Modern lifestyles result in ubiquitous daily exposures to a combination of environmental factors and mixtures of EDCs such as pesticides (e.g. DDT, DDE), herbicides, plasticizers, persistent organic pollutants (POPs), etc. that can accumulate in tissues and fluids.^46^ Experimental studies have shown that a variety of pesticides such as DDT/DDE, aldrin, dieldrin, etc. can interact with endocrine system components during critical periods of development and produce an equally varied spectrum of adverse developmental effects such as altered social skills, decreased intelligence, and reproductive difficulties or failures.^47^ Recent studies have found that EDCs not only affect reproductive and developmental health, endocrine function, but also adversely affect immune system function.^48^,^49^ It has been shown that EDCs exposure induces dysfunction of the immune system, which, in turn, has detrimental effects on metabolic health resulting in environmentally-induced diabetes and obesity. Endocrine disrupting chemicals can also change gene expression and epigenetic marks in the tissues where they accumulate and may act in a synergic way.^46^

Our findings may be in partial agreement with studies addressing adverse effects of pesticides on health. There are no populations completely unexposed to pesticides. The general population is exposed through pesticide residues present in food, water, indoor and outdoor air, soil and house dust, among other routes. Pesticide exposure is linked to diseases including cancer, hormone disruption, problems with reproduction and fetal development, asthma, allergies, and hypersensitivity.^45^,^50^–^51^

It is difficult to compare pesticide levels between occupational and environmental studies. Studies indicate increased health risks with occupational and accidental exposure to pesticides, including some respiratory diseases/symptoms.^50^

It is well known that among agrochemicals, OCPs are the most dangerous due to their persistency and highly lipophilic features. These compounds can be detected throughout the world in sediments and in the food chain. Humans are primarily exposed through diet.^23^ Even very low concentrations of pesticides and OCPs circulating in the environment and detected in agricultural products might have adverse effects on human health manifesting in different forms, the development of chronic respiratory diseases, and the increasing risk of asthma development.^26^,^44^,^50^–^51^ Taking into account the existing practice of pesticide use and the results of the present study, we may assume that OCPs found in soil and plant products could be associated to some extent with the observed pattern of respiratory morbidity.

There were some limitations to the present study. First, beyond chemical contamination of the environment with OCPs, other environmental factors such as air pollution with particulate matter and other chemicals, climate change factors and allergens, etc., could also play a role in respiratory disease dynamics. Furthermore, a more detailed analysis should take into account characteristics of the population such as age structure, smoking, and obesity, etc.

## Conclusions

The present study was carried out to study the levels of OCPs in soil and plant products in two areas in Armenia, Lori and Gegharkunik, to determine possible connections with respiratory diseases and asthma. The results showed that the ACh calculated for asthma tended to increase in Sevan and Gavar (Gegharkunik province). Alongside these findings, in Gegharkunik province the highest levels of AATC of studied OCPs were found in the soil samples from the Vardenis and Sevan/Gavar areas. These findings differed significantly from those recorded in Lori province, namely in the Stepanavan and Tashir areas (p = 0.01–0.0007). In plant products sampled from the Gegharkunik province, the AATC of OCPs was also significantly higher in comparison with Lori province (p = 0.001). To the best of our knowledge, our research is one of the few studies to analyze possible links between environmental exposure by organochlorine pesticides and respiratory diseases and asthma, particularly in Armenia.

Findings obtained on OCPs detected in different matrices and agricultural products sampled from studied areas in Armenia suggest a relationship to increased incidence of respiratory diseases and asthma. In Armenia, the health risks presented by endocrine-disrupting chemicals is of keen interest for specialists. The detection of DDT and HCH residues are evidence of their circulation in the environment. In Armenia, OCPs were commonly used in the past but have been removed from the market due to environmental and human health risks. Since the 1970s, the application of OCPs in the Republic of Armenia has been strictly prohibited.

Organochlorine pesticide residues detected in environmental matrices and plant products reflect the general practice of pesticide application in Armenia. This practice often does not comply with accepted guidelines due to lack of awareness among farmers on banned pesticides and possible adverse effects of pesticides. Active campaigns that educate and raise awareness among farmers about health and environmental risks are needed to mitigate the current situation by changing behavioral stereotypes and attitudes towards pesticides while promoting individual and community health.

A further comprehensive analysis will require epidemiological studies using questionnaires and interviews, starting at the community level. The present study was conducted at the regional level and based on official medical reports represents a first step for identifying possible associations between OCPs in Armenia and respiratory diseases. Further research is needed to study OCPs contamination dynamics and clarify their role in morbidity patterns, particularly with regard to respiratory morbidity.
